# 6,6′-Dieth­oxy-2,2′-[propane-1,3-diyl­dioxy­bis(nitrilo­methyl­idyne)]diphenol

**DOI:** 10.1107/S1600536810003430

**Published:** 2010-02-03

**Authors:** Li Wang, Yin-Xia Sun, Jun-Feng Tong

**Affiliations:** a School of Chemical and Biological Engineering, Lanzhou Jiaotong University, Lanzhou 730070, People’s Republic of China

## Abstract

The complete mol­ecule of the title compound, C_21_H_26_N_2_O_6_, is generated by a crystallographic twofold axis and adopts a *trans* configuration with respect to the azomethine group. The two benzene rings are almost perpendicular to one another, making a dihedral angle of 89.53 (3)°. In the mol­ecular structure, pairs of intra­molecular O—H⋯N hydrogen bonds generate two six-membered rings. The crystal structure is further stabilized by inter­molecular C—H⋯O hydrogen bonds, which link four adjacent mol­ecules into a network structure.

## Related literature

For background to salen-type bis­oxime compounds, see: Dong *et al.* (2007*a* ▶,*b* ▶; Dong & Duan, 2008 ▶). For the synthesis, see: Dong *et al.* (2008 ▶, 2009 ▶). For background to hydrogen bonding, see: Desiraju (1996 ▶). For hydrogen-bond motifs, see: Bernstein *et al.* (1995 ▶).
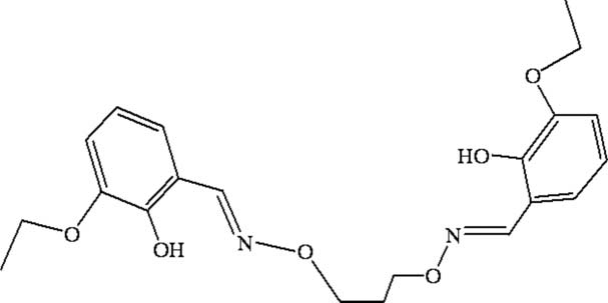


## Experimental

### 

#### Crystal data

C_21_H_26_N_2_O_6_
*M* *_r_* = 402.44
Orthorhombic, 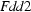

*a* = 25.292 (2) Å
*b* = 34.412 (3) Å
*c* = 4.7176 (5) Å
*V* = 4105.9 (7) Å^3^
*Z* = 8
Mo *K*α radiation
μ = 0.10 mm^−1^
*T* = 298 K
0.45 × 0.18 × 0.17 mm

#### Data collection

Bruker SMART 1000 CCD area-detector diffractometer
Absorption correction: multi-scan (*SADABS*; Sheldrick, 1996 ▶) *T*_min_ = 0.958, *T*_max_ = 0.984
5348 measured reflections
1031 independent reflections
817 reflections with *I* > 2σ(*I*)
*R*_int_ = 0.048

#### Refinement

*R*[*F* ^2^ > 2σ(*F* ^2^)] = 0.037
*wR*(*F* ^2^) = 0.090
*S* = 1.04
1031 reflections
133 parameters
1 restraint
H-atom parameters constrained
Δρ_max_ = 0.16 e Å^−3^
Δρ_min_ = −0.14 e Å^−3^

### 

Data collection: *SMART* (Siemens, 1996 ▶); cell refinement: *SAINT* (Siemens, 1996 ▶); data reduction: *SAINT*; program(s) used to solve structure: *SHELXS97* (Sheldrick, 2008 ▶); program(s) used to refine structure: *SHELXL97* (Sheldrick, 2008 ▶); molecular graphics: *SHELXTL* (Sheldrick, 2008 ▶); software used to prepare material for publication: *SHELXTL*.

## Supplementary Material

Crystal structure: contains datablocks global, I. DOI: 10.1107/S1600536810003430/hg2634sup1.cif

Structure factors: contains datablocks I. DOI: 10.1107/S1600536810003430/hg2634Isup2.hkl

Additional supplementary materials:  crystallographic information; 3D view; checkCIF report

## Figures and Tables

**Table 1:** Hydrogen-bond geometry (Å, °)

*D*—H⋯*A*	*D*—H	H⋯*A*	*D*⋯*A*	*D*—H⋯*A*
O2—H2⋯N1	0.82	1.92	2.636 (2)	145
C11—H11*C*⋯O1^i^	0.96	2.57	3.410 (3)	146

Symmetry code: (i) 
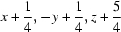
.

**Fig. 1.**
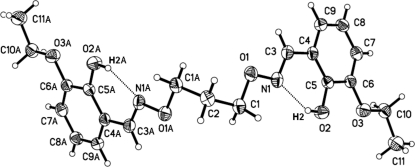
The molecule structure of the title compound with the atom numbering scheme [Symmetry codes for A atoms: -x+1/2, -y+1/2, z]. Displacement ellipsoids for non-hydrogen atoms are drawn at the 30% probability level.

**Fig. 2.**
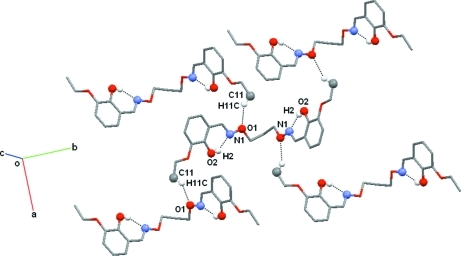
Digram showing the intramolecular O—H···N and intermolecular C—H···O hydrogen bonding interactions. Hydrogen atoms not involved have been deleated for clarity.

**Fig. 3.**
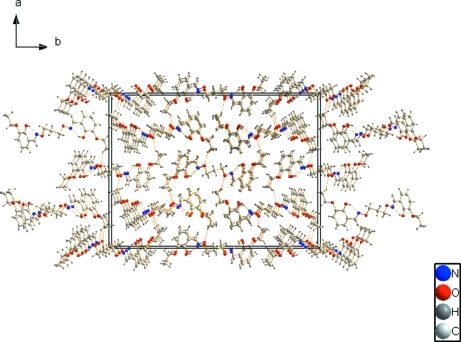
The packing digram of the title compound viewed along the b-axis.

## supplementary crystallographic information

### Comment

Salicylaldehyde and its derivatives are an important class of compounds which can be used in a variety of studies such as organic synthesis, catalysis, drug design, the spice industry and the life sciences (Dong *et al.*, 2007a; Dong & Duan, 2008). In the past few decades, continuing attention has been drawn to derivatives of the salicylaldehyde and their metal complexes for the investigation of luminescent properties which could be finely tuned by different substituent groups bonded to the phenolic ring (Dong *et al.*, 2008). In this paper, we report synthesis and X-ray structure of 6,6'-diethoxy-2,2'-[(1,3-propylene)dioxybis(nitrilomethylidyne)]diphenol, (I).

As shown in Fig. 1, the single-crystal structure of (I) is built up by only the C_21_H_26_N_2_O_6_ molecules, in which all bond lengths are in normal ranges. Two benzene rings (C4—C9) of molecule are perpendicular each other with a dihedral angle of 89.53 (3)°. There is a crystallographic twofold rotation axis passing through the middle point of the C—C—C unit. The molecule adopts a trans conguration in which two phenoldoxime moieties adopts an extended form, where the oxime and phenolic alcohols lie in trans positions relative to the C2 atom in the N—-O—CH_2_—CH_2_—CH_2_—O—N linkage. A pair of intramolecular O2—H2···N1 hydrogen bonds generate two six-membered rings, producing two S(6) ring motifs (Table 1, Fig. 1) (Bernstein *et al.*, 1995). Intermolecular C11—H11C···O1 hydrogen bonds (Desiraju *et al.*, 1996) link neighbouring molecules into a supermolecular structure (Fig. 2). Moreover, the packing diagram of the title compound shows a infinete netwok structure viewed along the b-axes (Fig. 3).

### Experimental

6,6'-Diethoxy-2,2'-[(1,3-propylene)dioxybis(nitrilomethylidyne)]diphenol was synthesized according to an analogous method reported earlier (Dong *et al.*, 2007b; Dong *et al.*, 2009). To an ethanol solution (4 ml) of 3-ethoxysalicylaldehyde(167.4 mg, 1.00 mmol) was added an ethanol solution (2 ml) of 1,3-bis(aminooxy)propylene (60.07 mg, 0.50 mmol). The mixed solution was stirred at 338 K for 5 h. The precipitate was filtered, and washed successively with ethanol and ether, respectively. The product was dried under vacuum to yield 160.3 mg of (I). Yield, 76.6%. m.p. 462.5-463.5 K. Anal. Calcd. for C_21_H_26_N_2_O_6_: C, 62.67; H, 6.51; N, 6.96. Found: C, 62.61; H, 6.40; N, 7.01.

Colorless needle-like single crystals suitable for X-ray diffraction studies were obtained after one weeks by slow evaporation from methanol solution of the title compound.

### Refinement

H atoms were placed in calculated positions and non-H atoms were refined anisotropically. The remaining H atoms were treated as riding atoms with distances C—H = 0.96 Å (CH_3_), 0.97 Å (CH_2_), 0.93 Å (CH), 0.82 Å (OH), and U_iso_(H) = 1.20 U_eq_(C), 1.50 U_eq_(O). In the absence of significant anomalous scatterers, Friedel pairs were merged before final refinement.

### Crystal data

**Table:**

C_21_H_26_N_2_O_6_	*F*(000) = 1712
*M**_r_* = 402.44	*D*_x_ = 1.302 Mg m^−^^3^
Orthorhombic, *F**d**d*2	Mo *K*α radiation, λ = 0.71073 Å
Hall symbol: F 2 -2d	Cell parameters from 1648 reflections
*a* = 25.292 (2) Å	θ = 2.9–25.3°
*b* = 34.412 (3) Å	µ = 0.10 mm^−^^1^
*c* = 4.7176 (5) Å	*T* = 298 K
*V* = 4105.9 (7) Å^3^	Needle-like, colorless
*Z* = 8	0.45 × 0.18 × 0.17 mm

### Data collection

**Table:**

Bruker SMART 1000 CCD area-detector diffractometer	1031 independent reflections
Radiation source: fine-focus sealed tube	817 reflections with *I* > 2σ(*I*)
graphite	*R*_int_ = 0.048
phi and ω scans	θ_max_ = 25.0°, θ_min_ = 2.0°
Absorption correction: multi-scan (*SADABS*; Sheldrick, 1996)	*h* = −30→29
*T*_min_ = 0.958, *T*_max_ = 0.984	*k* = −30→40
5348 measured reflections	*l* = −5→5

### Refinement

**Table:**

Refinement on *F*^2^	Primary atom site location: structure-invariant direct methods
Least-squares matrix: full	Secondary atom site location: difference Fourier map
*R*[*F*^2^ > 2σ(*F*^2^)] = 0.037	Hydrogen site location: inferred from neighbouring sites
*wR*(*F*^2^) = 0.090	H-atom parameters constrained
*S* = 1.04	*w* = 1/[σ^2^(*F*_o_^2^) + (0.051*P*)^2^] where *P* = (*F*_o_^2^ + 2*F*_c_^2^)/3
1031 reflections	(Δ/σ)_max_ < 0.001
133 parameters	Δρ_max_ = 0.16 e Å^−^^3^
1 restraint	Δρ_min_ = −0.14 e Å^−^^3^

### Special details

**Table:**

Geometry. All esds (except the esd in the dihedral angle between two l.s. planes) are estimated using the full covariance matrix. The cell esds are taken into account individually in the estimation of esds in distances, angles and torsion angles; correlations between esds in cell parameters are only used when they are defined by crystal symmetry. An approximate (isotropic) treatment of cell esds is used for estimating esds involving l.s. planes.
Refinement. Refinement of *F*^2^ against ALL reflections. The weighted *R*-factor wR and goodness of fit *S* are based on *F*^2^, conventional *R*-factors *R* are based on *F*, with *F* set to zero for negative *F*^2^. The threshold expression of *F*^2^ > σ(*F*^2^) is used only for calculating *R*-factors(gt) etc. and is not relevant to the choice of reflections for refinement. *R*-factors based on *F*^2^ are statistically about twice as large as those based on *F*, and *R*- factors based on ALL data will be even larger.

### Fractional atomic coordinates and isotropic or equivalent isotropic displacement parameters (Å^2^)

**Table:**

	*x*	*y*	*z*	*U*_iso_*/*U*_eq_	Occ. (<1)
N1	0.23158 (9)	0.17094 (6)	0.6113 (5)	0.0506 (6)	
O1	0.21889 (7)	0.20179 (5)	0.4290 (5)	0.0556 (5)	
O2	0.28824 (6)	0.11584 (5)	0.8533 (5)	0.0577 (6)	
H2	0.2826	0.1334	0.7399	0.087*	
O3	0.29360 (7)	0.05913 (6)	1.2148 (5)	0.0613 (6)	
C1	0.26566 (10)	0.21504 (7)	0.2881 (7)	0.0518 (7)	
H1A	0.2796	0.1948	0.1660	0.062*	
H1B	0.2926	0.2220	0.4255	0.062*	
C2	0.2500	0.2500	0.1155 (10)	0.0525 (10)	
H2A	0.2794	0.2571	−0.0059	0.063*	0.50
H2B	0.2206	0.2429	−0.0059	0.063*	0.50
C3	0.19084 (11)	0.15810 (7)	0.7412 (6)	0.0485 (7)	
H3	0.1580	0.1692	0.7050	0.058*	
C4	0.19489 (10)	0.12638 (7)	0.9451 (6)	0.0445 (7)	
C5	0.24271 (10)	0.10703 (7)	0.9928 (6)	0.0437 (7)	
C6	0.24500 (10)	0.07660 (7)	1.1909 (6)	0.0456 (7)	
C7	0.20034 (10)	0.06695 (8)	1.3422 (7)	0.0532 (7)	
H7	0.2021	0.0474	1.4782	0.064*	
C8	0.15281 (11)	0.08595 (8)	1.2947 (7)	0.0555 (8)	
H8	0.1228	0.0788	1.3960	0.067*	
C9	0.15006 (10)	0.11522 (8)	1.0988 (7)	0.0514 (7)	
H9	0.1181	0.1278	1.0674	0.062*	
C10	0.29937 (10)	0.02900 (8)	1.4204 (7)	0.0603 (9)	
H10A	0.2739	0.0085	1.3867	0.072*	
H10B	0.2939	0.0392	1.6100	0.072*	
C11	0.35463 (10)	0.01383 (9)	1.3886 (11)	0.0812 (12)	
H11A	0.3609	0.0072	1.1938	0.122*	
H11B	0.3590	−0.0088	1.5048	0.122*	
H11C	0.3793	0.0335	1.4467	0.122*	

### Atomic displacement parameters (Å^2^)

**Table:**

	*U*^11^	*U*^22^	*U*^33^	*U*^12^	*U*^13^	*U*^23^
N1	0.0629 (14)	0.0383 (12)	0.0506 (15)	−0.0003 (10)	−0.0073 (13)	0.0030 (13)
O1	0.0583 (11)	0.0451 (10)	0.0635 (14)	−0.0006 (8)	−0.0057 (10)	0.0129 (10)
O2	0.0507 (11)	0.0625 (12)	0.0599 (14)	0.0013 (8)	0.0034 (10)	0.0155 (12)
O3	0.0545 (10)	0.0622 (12)	0.0670 (15)	0.0057 (8)	−0.0027 (11)	0.0205 (12)
C1	0.0586 (17)	0.0438 (15)	0.0529 (18)	−0.0020 (12)	−0.0020 (15)	−0.0035 (15)
C2	0.060 (2)	0.051 (2)	0.046 (2)	−0.0072 (17)	0.000	0.000
C3	0.0545 (16)	0.0413 (15)	0.0499 (18)	0.0027 (12)	−0.0071 (15)	−0.0030 (15)
C4	0.0535 (15)	0.0354 (14)	0.0444 (17)	−0.0013 (11)	−0.0055 (14)	−0.0053 (13)
C5	0.0455 (14)	0.0428 (14)	0.0427 (17)	−0.0046 (11)	−0.0020 (13)	−0.0031 (14)
C6	0.0487 (14)	0.0417 (14)	0.0465 (17)	−0.0014 (11)	−0.0037 (13)	−0.0011 (14)
C7	0.0617 (17)	0.0478 (15)	0.0500 (18)	−0.0069 (12)	0.0014 (16)	0.0036 (15)
C8	0.0526 (15)	0.0552 (16)	0.059 (2)	−0.0083 (13)	0.0075 (15)	0.0010 (17)
C9	0.0460 (15)	0.0499 (15)	0.0582 (19)	0.0011 (11)	−0.0019 (14)	−0.0040 (16)
C10	0.0656 (19)	0.0518 (17)	0.063 (2)	−0.0042 (12)	−0.0133 (16)	0.0170 (17)
C11	0.0635 (19)	0.070 (2)	0.110 (3)	0.0007 (15)	−0.017 (2)	0.032 (3)

### Geometric parameters (Å, °)

**Table:**

N1—C3	1.278 (3)	C4—C5	1.399 (3)
N1—O1	1.403 (3)	C4—C9	1.400 (4)
O1—C1	1.431 (3)	C5—C6	1.405 (4)
O2—C5	1.361 (3)	C6—C7	1.377 (4)
O2—H2	0.8200	C7—C8	1.387 (4)
O3—C6	1.373 (3)	C7—H7	0.9300
O3—C10	1.427 (3)	C8—C9	1.369 (4)
C1—C2	1.506 (4)	C8—H8	0.9300
C1—H1A	0.9700	C9—H9	0.9300
C1—H1B	0.9700	C10—C11	1.500 (4)
C2—C1^i^	1.506 (4)	C10—H10A	0.9700
C2—H2A	0.9700	C10—H10B	0.9700
C2—H2B	0.9700	C11—H11A	0.9600
C3—C4	1.459 (4)	C11—H11B	0.9600
C3—H3	0.9300	C11—H11C	0.9600
			
C3—N1—O1	111.8 (2)	O3—C6—C7	125.9 (2)
N1—O1—C1	109.68 (18)	O3—C6—C5	114.7 (2)
C5—O2—H2	109.5	C7—C6—C5	119.4 (2)
C6—O3—C10	117.7 (2)	C6—C7—C8	120.9 (3)
O1—C1—C2	106.76 (19)	C6—C7—H7	119.5
O1—C1—H1A	110.4	C8—C7—H7	119.5
C2—C1—H1A	110.4	C9—C8—C7	120.0 (3)
O1—C1—H1B	110.4	C9—C8—H8	120.0
C2—C1—H1B	110.4	C7—C8—H8	120.0
H1A—C1—H1B	108.6	C8—C9—C4	120.7 (2)
C1^i^—C2—C1	114.5 (4)	C8—C9—H9	119.6
C1^i^—C2—H2A	108.6	C4—C9—H9	119.6
C1—C2—H2A	108.6	O3—C10—C11	106.3 (3)
C1^i^—C2—H2B	108.6	O3—C10—H10A	110.5
C1—C2—H2B	108.6	C11—C10—H10A	110.5
H2A—C2—H2B	107.6	O3—C10—H10B	110.5
N1—C3—C4	121.2 (2)	C11—C10—H10B	110.5
N1—C3—H3	119.4	H10A—C10—H10B	108.7
C4—C3—H3	119.4	C10—C11—H11A	109.5
C5—C4—C9	119.1 (3)	C10—C11—H11B	109.5
C5—C4—C3	121.5 (2)	H11A—C11—H11B	109.5
C9—C4—C3	119.3 (2)	C10—C11—H11C	109.5
O2—C5—C4	123.2 (2)	H11A—C11—H11C	109.5
O2—C5—C6	116.9 (2)	H11B—C11—H11C	109.5
C4—C5—C6	119.8 (2)		
			
C3—N1—O1—C1	−179.8 (2)	O2—C5—C6—O3	−0.9 (3)
N1—O1—C1—C2	−175.5 (2)	C4—C5—C6—O3	178.4 (2)
O1—C1—C2—C1^i^	67.48 (19)	O2—C5—C6—C7	178.7 (2)
O1—N1—C3—C4	−179.0 (2)	C4—C5—C6—C7	−1.9 (4)
N1—C3—C4—C5	−4.3 (4)	O3—C6—C7—C8	−178.2 (3)
N1—C3—C4—C9	175.1 (3)	C5—C6—C7—C8	2.2 (4)
C9—C4—C5—O2	179.9 (3)	C6—C7—C8—C9	−1.2 (4)
C3—C4—C5—O2	−0.7 (4)	C7—C8—C9—C4	−0.1 (4)
C9—C4—C5—C6	0.6 (4)	C5—C4—C9—C8	0.4 (4)
C3—C4—C5—C6	−180.0 (2)	C3—C4—C9—C8	−179.0 (3)
C10—O3—C6—C7	−2.1 (4)	C6—O3—C10—C11	176.8 (3)
C10—O3—C6—C5	177.5 (2)		

Symmetry codes: (i) −*x*+1/2, −*y*+1/2, *z*.

### Hydrogen-bond geometry (Å, °)

**Table:**

*D*—H···*A*	*D*—H	H···*A*	*D*···*A*	*D*—H···*A*
O2—H2···N1	0.82	1.92	2.636 (2)	145
C11—H11C···O1^ii^	0.96	2.57	3.410 (3)	146

Symmetry codes: (ii) *x*+1/4, −*y*+1/4, *z*+5/4.
